# Hospital Progress and Finance

**Published:** 1923-12

**Authors:** 


### HOSPITAL PROGRESS AND FINANCE.

### THE PROMISE OF VOLUNTARY INSURANCE.

It is possible that the insufficiency of beds from which the voluntary system suffers may benefit in the long run from the contribution schemes of recent foundation, though an early effect usually is to make the demand for beds more acute. Thus Mr. Philip K. Wake, the chairman of the Sheffield Royal Hospital, when lately referring to the 66 extra beds now available since the Princess of Wales's wing was opened, remarked that the contributors to the Joint Hospitals' Council scheme had urged the board of every hospital to increase its number of beds. From the Council, Mr. Wake went on, a handsome sum was already received, and now that the 60 beds had been added, a grant towards the cost of their maintenance would be applied for. The Sheffield scheme continues to grow, and there seems little doubt that eventually its adoption may become universal in the city. In this event sufficient money should be available, not merely to support the hospitals as they are, but to maintain the extensions so long overdue. If so, this will be a signal triumph of the Voluntary principle. When the unpopular Insurance Act was first passed, its more sober critics urged that it should have been voluntary. They were told that voluntary insurance schemes were inadequate and would remain so. But hospital provident schemes are a form of voluntary insurance, and if these eventually are able to support and extend the hospitals, the voluntary principle will have given State insurance a thorough lesson. Since it also works more smoothly than official schemes, the advantages may yet prove to be overwhelming.

### CONTRIBUTION SCHEMES AND NURSES' VISITS.

A CONFERENCE has been held between the organisers of the Sussex Hospital Provident Scheme and representatives of the Nursing Associations in the county, in the hope of securing co-operation between the two. Hitherto, the Provident Scheme has not touched nursing, and Major Crawley, who explained why it was so, said that a doctor's recommendation would be necessary before a nurse visited a contributor. Nursing associations, he went on, charged their members 1d. a week, and the committees set up to raise funds in villages could, he thought, supply 2d., where a member was unable to afford the weekly contribution of 3d. that membership of the Provident Scheme involves. It was finally agreed that a subscription of 4d. a week would make co-operation between the two bodies possible, and would give to contributors the right to hospital treatment, and to nurses' visits in their homes. A resolution recommending the plan to the Nurses' Associations was carried, since the alternative of a fixed charge for each visit from a nurse was not approved. The Provident Scheme has now 5,350 members. If these already find 3d. a week as much as they can pay, the additional penny may be difficult. At present the nursing associations everywhere rely as a rule on donations to make good their annual deficiency. These donors presumably will appreciate the advantages of co-operation, and continue their support. More may be needed, since these useful schemes generally add to the number of applicants for assistance.

### SECOND CLASS PRIVATE PATIENTS.

THE County Mental Hospital at Lancaster is receiving second-class private patients at a charge of 24s. 6d. a week. These are classified as private patients, and may wear their own clothes, but otherwise are accommodated in the same division as patients chargeable to the Union. The classification has been made because the committee of visitors found that it was desired by the families of those who could not afford the full fees for private patients, and yet were able and willing to pay the full cost of maintenance and the proportion incurred on the capital account. The guardians throughout the county have been informed of this, that the advantages offered may be known.

### BEDS FOR THE CONSULTING STAFF?

THE first introduction of a rule requiring hospital surgeons to retire at a certain age sometimes causes searchings of heart both to surgeons and hospital committees. A typical instance has occurred in connection with the Royal Portsmouth Hospital, where, because of the relatively recent rule, the senior honorary surgeon has had to retire although, as it happens, he has served no more than seventeen years in the office of senior surgeon, a period virtually shorter than that of any senior surgeon since the hospital was founded. By way of compensation, since the senior surgeon is understood to have devoted exceptional time to the hospital since he retired from general practice, the governors have proposed that three or six beds should be placed under him for three years, provided that he remains a consulting surgeon, and that this concession should be made only as an exception and not be regarded as a precedent. Opinions were divided and a further conference with the honorary staff has taken place. Since these lines will probably be in print before the decision has reached us, we defer comment. The simple question really is—should or should not the agreed age-limit be decisive ?

### LOCAL OR COUNTY ISOLATION HOSPITALS?

THE controversy that arises whenever a new isolation hospital is under discussion is notorious, but a new point emerges from the situation created by the conference of local authorities at Norwich on the matter. By the time that these lines are in print the delegates will have met, after consulting their councils, with power to vote no less than to discuss the issue. The crux of the difficulty is whether or not there should be a central isolation hospital for the county. The local Councils, who have often provided or united with other bodies to provide a local isolation hospital, are nervous lest they should be saddled with an additional expense if a central isolation hospital be established. The local Medical Officers of Health have hitherto naturally confined their attentions to local needs, and their recommendations have had to meet the traditional fears of local people. Nevertheless, a central hospital should prove more economical in the long run than several local institutions. So we are confronted with the ridiculous position of a sudden local desire to maintain the institutions that have been so reluctantly supplied or supported in the past. The right solution appears to be one that will demand no more from each local authority than the cost of maintaining a local hospital, while securing a central institution at an eventual economy of expense.

### MAYORS AND THE SECOND SCIENTIFIC EXHIBITION.

The Scientific Novelties Exhibition is to be repeated. It will be open from December 29 to January 9, inclusive, between the hours of 2 and 9 p.m., at King's College. This is the best proof of the interest aroused last year, when a substantial sum was raised for the London hospitals. The proceeds will go to King Edward's Hospital Fund. Lectures, experiments and demonstrations or screen pictures will again be given, and already many distinguished men of science have promised their support. The decision is a useful one, not only for its immediate result. It is part of an awakening to the need for the regular instruction of the public in scientific and medical matters. We therefore hope that it will be attended by the Mayors of London boroughs, to encourage all of them to follow the few pioneers in making their own town halls the centre of similar instruction, which the success of several "Health Weeks" held with their co-operation is justifying. Medical Officers of Health in London will respond much more readily to the trouble involved by these "Weeks" if they are encouraged to regard them as the beginning of a permanent educational campaign with the local town hall for its appropriate centre.

### THE LEEDS HOSPITAL FUND.

Mr. T. F. Braime, that good friend of the Leeds voluntary hospitals, whose fertile brain has begotten several useful schemes on their behalf, has now given news of the progress of his latest. The city was divided into twenty sections and the results in four sections analysed, the number of firms in each calculated, the number of firms subscribing deducted, and the remainder, by degrees, approached personally. The total number of firms was 831, of whom 123 subscribed £732 to begin with. Now one hundred of these firms, who contributed £513 last year, contribute £1,720. There are altogether 4,133 firms in the twenty sections, which under the old scheme subscribed £2,437, or about ten shillings a firm. To-day, as we have seen, one-fortieth of the number of firms are raising over half of the previous grand total from them all. If all the city firms followed suit, a sum of £70,000 would be raised for the Voluntary Hospital Fund. The improvement made is attributed by Mr. Braime to personal application by others and himself interested in the Fund. Haphazard action is now seen to involve much waste. By definition and dissection a great city can be made to discover its individuality, and hospital needs brought personally before almost every one of its citizens.

### A DORSET MENTAL CLINIC.

The members of the South-Western division of the Medico-Psychological Association held their annual meeting at the Devon Mental Hospital, Axminster, when, after inspecting the wards of the workshops, Dr. G. Peachell, Medical Superintendent of the Dorset Mental Hospital, described the out-patient clinic which was started there a year ago. By co-operation with the Dorset County Hospital, the latter was paid a fixed sum yearly for the use of the out-patient room as a clinic. During the year a large number attended, and the suggested lesson was that if this was the result in Dorset, greater results should follow from clinics established in centres like Exeter. The sense of the meeting was that these clinics should be not the exception but the rule, and much was hoped from the Mental Treatment Bill, whereby early cases could be received without certification. In the military hospitals for nerve cases the rate of recovery was stated by the chairman, Dr. Eager, to have been 56 per cent. of early cases, whereas the average recovery rate in the civil hospitals was not more than 32. The feeling in favour of a change in the law was unanimous, and this almost certainly reflects the general medical opinion in the country. It remains to be seen what the new House of Commons will do.

### THE NEW EAST SUSSEX HOSPITAL.

If the authorities of the East Sussex Hospital at Hastings were unfortunate in the day of the formal opening of their new premises, they are extremely fortunate in a far more permanent matter—the site of the hospital. The old quarters were situated directly on the sea front and were too accessible both to noise and dust; the present building stands high above the White Rock Gardens, with an excellent view over the sea and open to the salt-laden breezes. In spite of the heavy rain which fell throughout the chosen day at the end of October, Princess Alice, Countess of Athlone, kept her promise to declare the new premises open, and spent an hour or so inspecting the wards and giving much pleasure to both staff and patients. The hospital is built in three blocks with a paved courtyard between each, and with large windows and balconies to permit of the patients enjoying every gleam of sunshine. There is accommodation for 106 men, women and children, with five large wards and several smaller ones, the latter being devoted to the nursing of ophthalmic, isolation and gynæcological cases. The hospital serves a large area, including the Boroughs of Hastings and Bexhill and the surrounding districts of East Sussex, and its beds are always occupied.

